# Preliminary Evaluation of a Web-Based Psychological Screening Tool in Adolescents Undergoing Minimally Invasive Pectus Surgery: Single-Center Observational Cohort Study

**DOI:** 10.2196/mental.9806

**Published:** 2018-05-31

**Authors:** Davina Wildemeersch, Lisa Bernaerts, Michiel D’Hondt, Guy Hans

**Affiliations:** ^1^ Antwerp University Hospital Edegem Belgium

**Keywords:** mental health, telemedicine, pectus carinatum, funnel chest

## Abstract

**Background:**

Preoperative anxiety and depression are predominant risk factors for increased postoperative pain. Thoracic wall deformities in adolescents often cause low self-esteem, which contributes to psychological concerns. Several studies have suggested a relationship between preoperative mental health support and enhanced recovery after surgery.

**Objective:**

This study investigated the validity of screening questionnaires concerning psychological trait and state characteristics via a patient-specific online platform.

**Methods:**

Patients scheduled for elective pectus surgery between June 2017 and August 2017 were invited to participate in clinical interviews and online self-report questionnaires. All patients were recruited in the Anesthesiology Department, Antwerp University Hospital, Belgium. This single-center observational cohort study was performed in accordance with the ethical standards of the International Council for Harmonisation–Good Clinical Practice guidelines and the Declaration of Helsinki after obtaining study approval by the Institutional Review Board and Ethics Committee of the Antwerp University Hospital, Belgium (study identifier: 17/08/082). An online preoperative psychological inventory was performed using the Rosenberg Self-Esteem Scale, Hospital Anxiety and Depression Scale, and State-Trait Anxiety Inventory. Postoperatively, pain intensity and interference were assessed using the Multidisciplinary Pain Inventory, Coping With Pain Questionnaire, and numeric pain rating scale assessment. Patient satisfaction of the Web-based platform was evaluated.

**Results:**

A total of 21 adolescent patients used our Web-based psychological perioperative screening platform. Patients rated the mobile phone app, usability, and accessibility of the digital platform as good or excellent in 85% (17/20), 89% (17/19), and 95% (20/21) of the cases, respectively. A total of 89% (17/19) of the patients rated the effort of generating answers to the online questionnaires as low. The results from the completed questionnaires indicated a strong negative correlation between self-esteem and the anxiety trait (*R*=–0.72, *P*<.001) and overall anxiety characteristics (*R*=–0.49, *P*=.04). There was a positive correlation between depressive and anxiety characteristics and the anxiety trait (*R*=0.52, *P*=.03 and *R*=0.6, *P*=.02, respectively) measured by the online self-report questionnaires. Moreover, preoperative anxiety was positively correlated with postoperative pain interference (*R*=0.58, *P*=.02). Finally, there was a negative correlation between self-esteem and pain interference (*R*=–0.62, *P*=.01).

Conclusions*:* Perioperative screening of psychological symptoms and trait characteristics with specific treatment, if necessary, could further improve postoperative pain and overall health status. Research on eHealth technology, even for psychological patient care, is rapidly increasing.

**Trial Registration:**

ClinicalTrials.gov NCT03100669; https://clinicaltrials.gov/ct2/show/NCT03100669 (Archived by WebCite at http://www.webcitation.org/6zPvHDhU5)

## Introduction

Pectus excavatum and carinatum occur in 1 of 400 to 1000 children, with a 4:1 male-to-female predominance [1]. Many patients experience aesthetic challenges and even a compromised self-esteem during the vulnerable phase of puberty. Surgery is more often planned for aesthetic reasons than a necessary correction due to compression of underlying organs. Although minimally invasive repair of pectus (MIRP) has become common practice because of surgical stress response reduction, less blood loss, and a smaller incision, it still remains associated with severe postoperative pain. Moreover, the intensity of postoperative pain following MIRP has been shown to be the overriding factor in a patient’s perception of the quality of the postoperative period. The fact that many adolescents experience moderate to severe pain for the first time and develop a new dependence on their parents further contributes to their decreased well-being after the surgical procedure. Many investigators have shown that preoperative psychosocial factors such as anxiety further increase postsurgical pain [2-4].

Recently, several authors assessed quality of life and self-esteem following surgical pectus repair [5-7]. Not surprisingly, adolescents with a chest wall deformity have lower self-esteem and higher anxiety or even depressive characteristics than healthy controls. Moreover, children and parents experience surgery as a stressful period and often feel underprepared for the operation, postoperative pain, and recovery. Many of them reported an interest in perioperative psychosocial screening. Previous research by Rabbitts et al [8] showed that health care providers agree that families would benefit from enhanced coping skills. Therefore, investigators have proposed a flexible screening tool to examine anxiety and dysfunctional coping strategies in children undergoing major surgery [8].

Little research has been conducted on the influence of preoperative psychological questionnaires on postoperative pain via eHealth services. With such services, patients and their relatives can complete questionnaires when and where they want, making participation less demanding. Even more, caregivers can introduce mental health screening before surgery as part of the surgical care.

The primary aim of the study was to develop and implement a Web-based patient platform for preoperative psychological yellow flag screening and early identification of risk factors for subacute or persistent postoperative pain. In addition, the applied screening battery was evaluated for usefulness in adolescents undergoing elective pectus surgery and feasibility for online questionnaire completion.

Psychological variables and their relationship with postoperative outcome parameters such as persistent, subacute pain were assessed. Finally, self-esteem was evaluated, being an important indirect factor contributing to persistent pain via the development of anxiety, depression, or maladaptive coping strategies.

## Methods

### Recruitment

**Figure 1:**
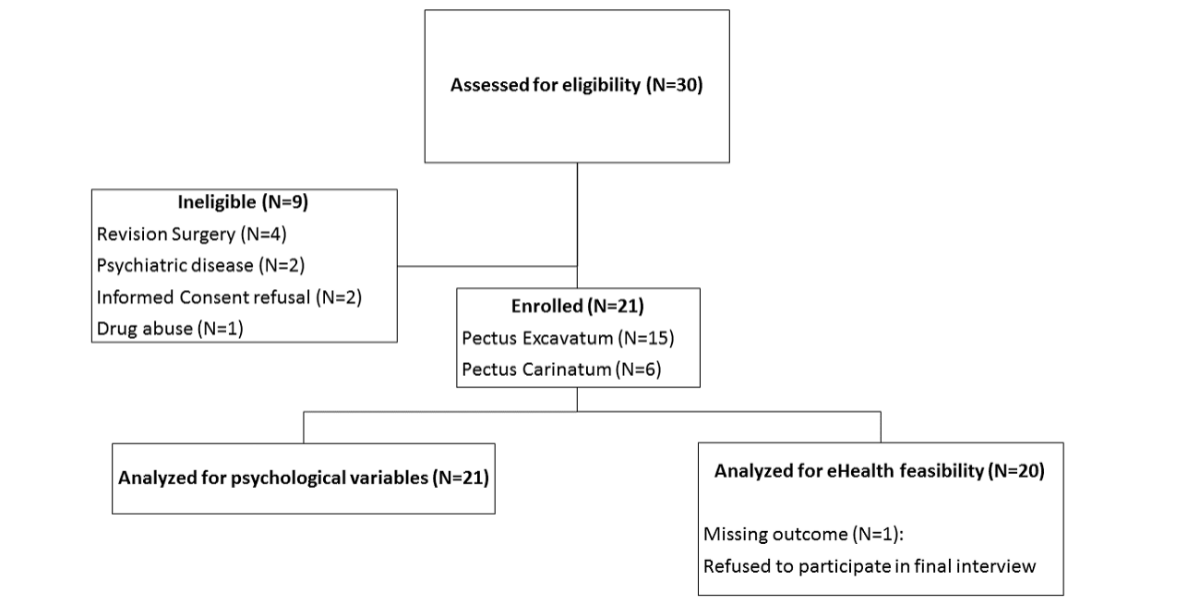
Flow diagram of patient screening and study inclusion during the summer holidays of 2017.

A total of 22 patients were scheduled for elective pectus surgery during summer holidays (June to August 2017) and were invited for clinical interviews and to complete online self-report questionnaires. All patients were recruited in the Anesthesiology Department, Antwerp University Hospital, Belgium (Figure 1). This single-center observational cohort study was performed in accordance with the ethical standards of International Council for Harmonisation–Good Clinical Practice and the Declaration of Helsinki after obtaining study approval by the Institutional Review Board and Ethics Committee of the Antwerp University Hospital, Belgium (study identifier: 17/08/082). Patients with a history of psychiatric disease, chronic opioid use (more than 3 months), or revision surgery were excluded. No single patient reported clinically relevant preoperative pain symptoms.

**Figure 2:**
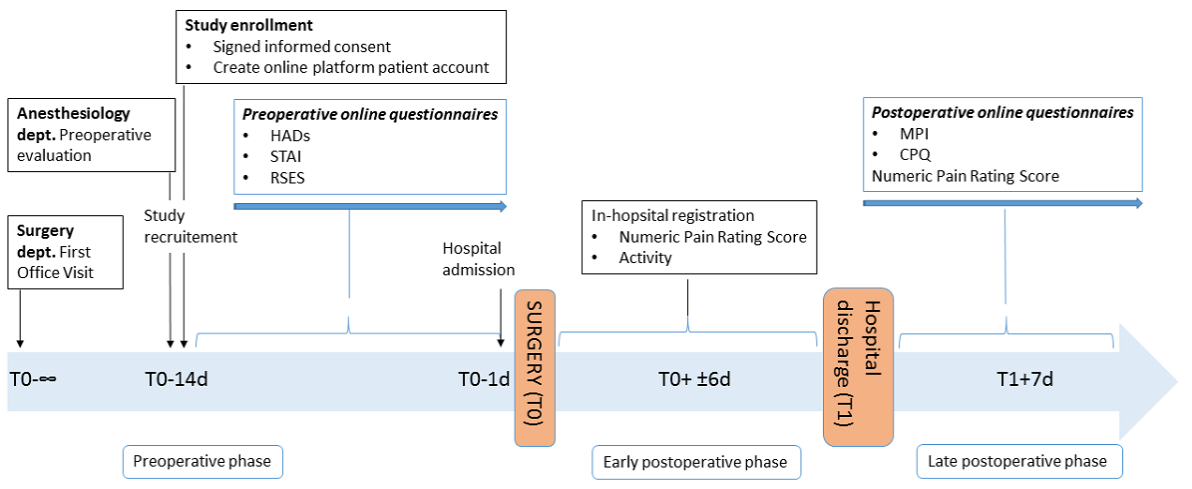
Study design.

Analyses of variance revealed no significant differences between pectus excavatum and pectus carinatum patients with respect to age or body mass index. The Haller index for defining the severity of the deformity in pectus excavatum patients based on computed tomography (CT) varied from 3.00 to 7.00 (mean 3.59 [SD 1.47]; median 3.00 [95% CI 2.24-4.95]) in the 50% of pectus excavatum patients who had CT performed. The mean age of the subjects was 14.82 (SD 1.30) years, and the majority of the participants (20/21, 95%) were men; 90% (19/21) were not the only child in the family, and 52% (11/21) had a high education level (general secondary education–high school). Figure 2 shows a flowchart of the study.

### Web-Based Platform

**Figure 3:**
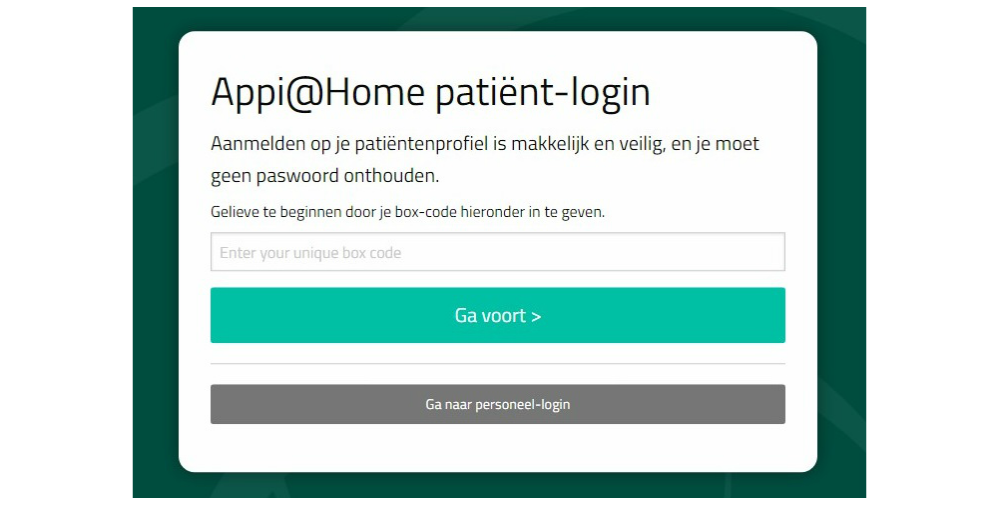
Appi@home online platform—patient view.

**Figure 4:**
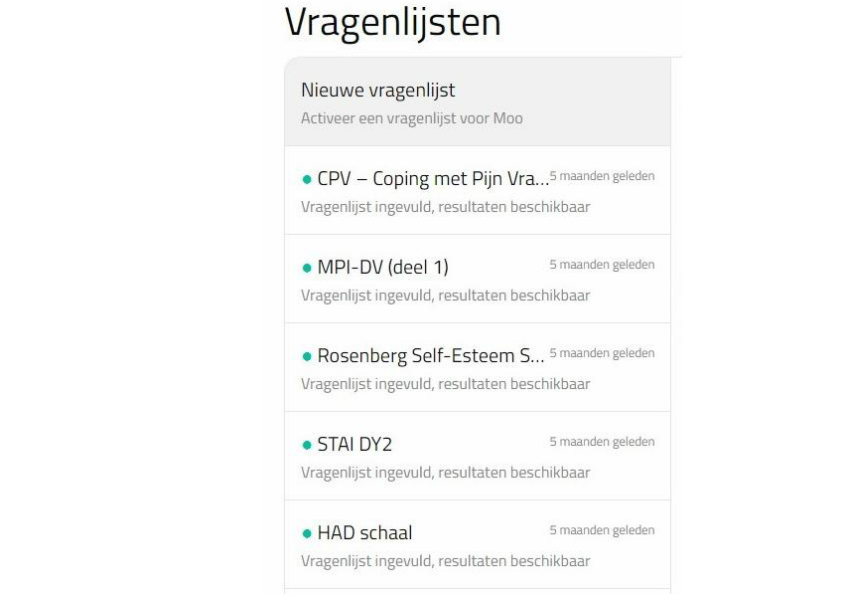
Appi@home online platform—provider view listing completed questionnaires (Vragenlijsten).

To provide patients with an individualized approach, we developed an electronic medical record coupled with a set of questionnaires. The Antwerp Personalized Pain Initiative app (Appi@home, see Figures 3 and 4) supports an innovative approach by offering an online platform. Patients are provided with a unique code that allows them to fill out the preselected questionnaires. In addition, the patient becomes an active participant in the global preventive and further therapeutic approach, if necessary.

### Preoperative Psychological Assessments

#### State-Trait Anxiety Inventory

The State-Trait Anxiety Inventory (STAI) Form Y is an instrument used to assess state and trait anxiety. State anxiety is defined as fear, nervousness, and discomfort temporarily induced by situations perceived as dangerous or threatening in which the autonomic nervous system is activated. State anxiety can vary in intensity and change over time. Trait anxiety involves rather stable individual differences in the predisposition to experiencing fear, stress, and discomfort. People with high trait anxiety characteristics will experience certain situations as more threatening or dangerous than people with low trait anxiety. In this study, the Dutch version (STAI-version-DY-2) was used. This 20-item scale is designed to assess pervasive feelings of trait anxiety. Items are rated by respondents on a 4-point Likert-type scale. Higher scores indicate higher levels of anxiety, and norm tables are available for different groups. The STAI-version-DY-2 has demonstrated acceptable internal consistency (alpha>.85) and 1-month test/retest reliability (*r*>.70) in adolescents, healthy adults, and military samples [9]. Van Der Ploeg et al [10] developed a Dutch translation [11].

#### Hospital Anxiety and Depression Scale

The Hospital Anxiety and Depression Scale (HADS) has been developed to detect states of depression and anxiety in a hospital setting. It assesses core components of anxiety and depression without involving physical complaints. The questionnaire has 2 subscales, anxiety and fear, and each subscale consists of 7 items. Higher scores indicate more emotional complaints. Cutoff scores are available for quantification. For each subscale, a score of 8 or greater is associated with possible anxiety or depression. A score of 11 or greater is associated with probable anxiety or depression. The questionnaire was developed as a screening tool and can exclude but not assess emotional disorders [12,13]. The basic psychometric properties of the HADS as a self-rating instrument should be considered quite good in terms of factor structure, intercorrelation, homogeneity, and internal consistency [14]. Spinhoven et al [15] validated a Dutch version that was used in this study.

#### Rosenberg Self-Esteem Scale

The Rosenberg Self-Esteem Scale (RSES) is a self-report measure for self-esteem containing 10 items that was constructed for the investigation of a person’s feelings about themselves in terms of self-confidence and intrinsic value. Self-esteem is an important measure for screening problems of social adaptation and predicting mental health problems. Items are rated by respondents on a 4-point Likert-type scale [16]. We used 2 scoring procedures for optimal interpretation of our results. The total score ranges from 0 to 30 according to the first procedure and from 10 to 40 according to the second procedure. The higher the total scores, the higher the level of self-esteem. Franck et al [17] developed the Dutch translation and evaluated the psychometric properties. The results showed high internal consistency and high congruent validity. Their findings support the usefulness of the Dutch RSES as a measure of self-esteem [17].

### Postoperative Psychological Assessments

#### Multidimensional Pain Inventory

Kerns et al [18] applied cognitive behavioral concepts on chronic pain and developed the (West Haven–Yale) Multidimensional Pain Inventory (MPI). This questionnaire assesses different pain-relevant aspects. The subjective characteristic of pain and the consequences on different aspects of the patient’s life are the main objectives of the questionnaire [18]. Lousberg et al [19] developed a Dutch version of the questionnaire (MPI-DVL). The MPI-DVL consists of 61 items, ordered in 3 parts. The first part, used by the authors to assess the psychosocial aspects of pain, consists of 5 subscales: pain severity, interference, life control, affective distress, and social support. Items are rated by respondents on a 7-point Likert-type scale. The authors evaluated the psychometric properties of the Dutch version, and their results showed good reliability and validity [19]. In this study, the first 2 subscales (pain severity and interference) are used for data analyses.

#### Coping With Pain Questionnaire

The Coping Strategy Questionnaire (CSQ) is an instrument developed to assess the coping strategies people use when experiencing pain. Research has shown that people develop their own coping style resulting from past experiences with pain and a general coping style for difficult situations. This instrument contains 44 items designed to evaluate 8 strategies for coping with pain (reinterpreting pain sensations, using coping self-statements, ignoring sensations, diverting attention, praying/hoping, catastrophizing, increasing behavioral activities, and exhibiting pain behaviors). The perceived effectiveness of the coping efforts was assessed with 2 items: control over pain and the ability to decrease pain [20]. Spinhoven et al [21] developed the Dutch version of the CSQ, the Coping With Pain Questionnaire (CPQ), which is slightly different. The CPQ contains 44 items in 8 subscales (diverting attention, reinterpreting pain sensations, using coping self-statements, ignoring pain sensations, praying/hoping, catastrophizing, increasing behavioral activities, and perceiving control over pain). The respondent answers questions on a visual analog scale (VAS) for the CPQ instead of a 7-point Likert-type scale (for the CSQ). The respondents indicate how often they use a specific coping behavior by putting a line on a 10-cm–long line with end points defined as “I never do that” and “I always do that.” The psychometric properties of the instrument are good [21]. CPQ active and passive coping indices were calculated according to the method described by Soares and Grossi [22] and Nicholas et al [23]. The scores of 5 subscales (diverting attention, reinterpreting pain sensations, coping self-statements, ignoring pain sensations, and increasing behavioral activities), which reflect active coping, were calculated to determine an active coping index. Two scales (catastrophizing, praying/hoping) that refer to passive coping were used to create a passive coping index. The subscale that assessed perceived control over pain was the self-efficacy index [22,23].

### Numerical Rating Scale

The numerical rating scale (NRS) is an 11-point scale used for pain assessment. Self-report by a patient is considered the gold standard for pain intensity measurement. Caregivers familiar with communicating with patients in pain asked the patient how much pain they had suffered from in the previous 24-hour period. All patients were educated in pain rating, where 0 represents “no pain” and 10 represents “the worst pain possible,” using whole numbers. The mean score after the first 5 postoperative days was calculated [24]. Patients continued the pain intensity registration through the platform until completion of the postoperative questionnaires, 7 days after hospital discharge.

### Daily Activity and Patient Mobility

Patients were assessed according to their mobility and daily activity by the attending physiotherapist. Every patient was given a daily score based on the exercise executed as part of the rehabilitation process after surgery during hospitalization. Scores ranged from 1 (exercise in the supine position), 2 (sitting), 3 (standing), to 4 (walking).

### Statistical Analysis

A paired sample *t* test was used to assess differences in RSES bifactor questionnaire scoring after data normality assessment with the Shapiro-Wilk test. Associations between questionnaire scores were determined with a Spearman correlation coefficient. Statistical analyses were performed with SPSS Statistics software (IBM Corp). Statistical significance was considered when *P*<.05.

## Results

### Patient Demographics and Questionnaire Responses

The demographic patient characteristics are presented in Table 1. Eighteen adolescents completed the preoperative questionnaires, and 16 fully completed the postoperative questionnaires (Table 2). Furthermore, from the raw CPQ data, coping subscales were calculated to score the pectus patients on 3 coping categories (active coping strategy, passive coping strategy, and self-efficacy).

**Table 1:** Sociodemographic characteristics (N=21).

Characteristic	Result
Type of deformity, n, PE^a^: PC^b^	15:6
Gender, n, male: female	20:1
Age, years, mean (SD)	14.81 (1.33)
Height, cm, mean (SD)	173.67 (8.88)
BMI^c^, kg/m^2^, mean (SD)	18.44 (2.03)

^a^PE: pectus excavatum.

^b^PC: pectus carinatum.

^c^BMI: body mass index.

**Table 2:** Anxiety and depression characteristics, self-esteem rating, multidimensional pain questionnaire results, and coping with pain evaluation via eHealth technology.

Questionnaire		Score, mean (SD)
HADS^a^ fear		6.11 (3.27)
HADS depression		3.50 (2.81)
STAI^b^		37.94 (6.88)
RSES^c^		21.56 (3.55)
**MPI^d^**		
	Pain severity	1.88 (0.78)
	Pain interference	3.20 (0.69)
**CPQ^e^ (raw data)**		
	Diverting attention	3.88 (2.05)
	Reinterpreting pain sensation	23.29 (12.30)
	Catastrophizing	9.59 (8.42)
	Ignoring pain sensation	25.18 (12.69)
	Praying/hoping	23.47 (14.64)
	Coping self-statements	38.94 (12.12)
	Increasing behavioral activities	21.71 (9.84)
	Perceiving pain control	11.59 (4.65)
**CPQ subscale**		
	Active coping score (raw data)	23.52 (7.41)
	Passive coping score (raw data)	16.53 (9.22)
	Self-efficacy score (raw data)	11.59 (4.65)

^a^HADS: Hospital Anxiety and Depression Scale.

^b^STAI: State-Trait Anxiety Inventory.

^c^RSES: Rosenberg Self-Esteem Scale.

^d^MPI: Multidimensional Pain Inventory.

^e^CPQ: Coping With Pain Questionnaire.

### Detailed Questionnaire Data

#### Hospital Anxiety and Depression Scale

The HADS fear subscale indicated the presence of an anxiety disorder. The overall mean score was 6.11 (SD 3.27). The mean score ranged from 0 to 7, which indicated the absence of anxiety states prior to surgery. Thirteen patients scored between the range of 0 to 7 (no anxiety), 3 patients scored between the range of 8 to 10 (possible anxiety), and 2 patients scored in the range of 11 or higher (probable anxiety).

The HADS depression subscale indicated the presence of a depressive disorder. The overall mean score was 3.50 (SD 2.81). This mean score ranged from 0 to 7, which indicated the absence of depressive states prior to surgery. Sixteen patients scored between the range of 0 to 7 (no depression), and 2 patients scored between the range of 8 to 10 (possible depression).

#### State-Trait Anxiety Inventory

The DY-2 version of the STAI measured trait anxiety. The overall mean score of the study sample was 37.94 (SD 6.88). Compared with available data on controls (normal group of male military recruits approximately 18 years old), the overall mean score was in decile 6 indicating a mean level of anxiety.

#### Rosenberg Self-Esteem Scale

The RSES is a measure of global self-esteem. The mean score of the overall patient sample was 21.56 (SD 3.55) and was above the theoretical midpoint of 15. No single patient scored beneath this cutoff. The results can be compared with the data from the study by Schmitt and Allik [25], in which self-esteem levels were compared across 53 nations. The mean scores of this study sample were above the Belgian mean level of 19.66 (SD 5.28). The results of this study sample were higher than the average level of global self-esteem.

#### Multidimensional Pain Inventory

The MPI measured different pain-relevant aspects. The mean score of the study sample was compared with available normative data (mean and standard deviation) of the International Association for the Study of Pain Primary Site: Thoracic Region [18]. The overall mean pain severity score was 1.88 (SD 0.78), which was lower than the mean score of the normative sample (5.01 [SD 0.82]). The overall mean pain interference score was 3.20 (SD 0.69), which was lower than the mean score of the normative sample (5.01 [SD 0.80]).

#### Coping With Pain Questionnaire

The CPQ assessed different pain coping strategies. The mean raw subscale scores were compared with those of a normal group of patients with chronic low back pain or neck pain. The decile scores are written in parentheses below. The overall mean diverting attention score was 23.29 (SD 12.30; decile 5). The overall mean reinterpreting pain sensation score was 8.47 (SD 6.99; decile 2). The overall mean catastrophizing score was 9.59 (SD 8.42; decile 2). The overall mean ignoring pain sensation score was 25.18 (SD 12.69; decile 4). The overall mean praying/hoping score was 23.47 (SD 14.64; decile 6). The overall mean coping self-statements score was 38.94 (SD 12.12; decile 6). The overall mean increasing behavioral activities score was 21.71 (SD 9.84; decile 3). The overall mean perceiving pain control score was 11.59 (SD 4.65; decile 7). Note that these scores represent the pain coping ability of the study sample. The mean postoperative pain (day 1 to day 5) was low (mean NRS 1.89, mean MPI pain severity 1.88), reflecting the need to develop strategies to cope with pain.

### Postoperative Pain

As shown in Table 3, all included patients received a postoperative evaluation score involving pain assessment (NRS) during hospital admission and a reassessment before postoperative questionnaire completion.

### eHealth Technology

The primary variable was a patient’s global assessment of the feasibility for the mobile phone app, internet platform, and accessibility of the questionnaires (using a 4-point categorical scale where 1=poor, 2=fair, 3=good, and 4=excellent). Twenty patients rated the eHealth implementation at the final interview after questionnaire completion.

Secondary end points included the time burden for questionnaire completion (using a 5-point categorical scale, where 1=low burden, 2=rather low, 3=average, 4=rather high, and 5=high) and response burden after a single reminder of the importance of questionnaire completion.

Patients rated the mobile phone app, individual online platform usability, and accessibility as good or excellent in 85% (17/20), 89% (17/19), and 95% (20/21) of responses, respectively. No individual scored the usability or accessibility as poor. Regarding the time burden assessment, 67% (12/18) indicated a (rather) low effort for questionnaire completion, and 22% (4/18) mentioned an average effort was required. Overall, 76% (16/21) of the patients were able to complete the online questionnaires within the imposed deadline.

### Correlations

#### Preoperative Psychological Screening Tool

Assessing the usefulness of the online implemented questionnaires, correlations have been calculated. The results (see Table 4) showed a strong negative correlation between self-esteem (RSES) and anxiety characteristics (HADS anxiety) and between self-esteem and anxiety trait scores (STAI). Furthermore, there was a positive correlation between STAI and anxiety characteristics and depression symptoms (HADS anxiety and depression).

#### Pain Measurement, Inpatient Versus Outpatient Evaluation

The study findings showed a low positive correlation between the mean pain scores for the first 5 days after surgery and the pain severity scores measured with the postoperative questionnaire after hospital discharge (*R*=0.35, *P*=.18), although the differences were not significant. No correlation was found between daily activity scores and pain severity and pain interference.

#### Preoperative Psychological Screening Tools and Postoperative Outcome Measures (Pain and Coping Characteristics)

Finally, the results demonstrated a negative but nonsignificant correlation between self-esteem and pain interference (*R*=–0.62, *P*=.14; Table 5). There was a positive correlation between present anxiety characteristics and passive coping behavior (*R*=0.55, *P*=.03; Table 6) and anxiety trait and pain interference (*R*=0.58, *P*=.02). A clearly positive correlation was noted between postoperative pain score after hospital discharge and pain severity assessed by the MPI (*R*=0.62, *P*=.02).

No significant correlation was found between preoperative psychological screening questionnaires and mean postoperative pain scores or between coping and pain (passive coping index vs pain, *R*=0.26, *P*=.32; catastrophizing vs pain, *R*=0.04, *P*=.87).

**Table 3:** Pain rating scores up to 5 days after surgery and highest mean pain score before postoperative questionnaire completion (first week after hospital discharge).

Numerical rating scale for pain assessment	Score, mean (SD)
Postoperative day 1	1.36 (1.43)
Postoperative day 2	2.10 (2.00)
Postoperative day 3	1.91 (1.38)
Postoperative day 4	2.71 (1.79)
Postoperative day 5	2.09 (1.15)
First 5 postoperative days	1.89 (0.82)
Highest mean score before questionnaire completion	3.13 (1.83)

**Table 4:** Correlation between the preoperative psychological dimensions.

Variables	Self-esteem		Depressive characteristics		Anxiety characteristics		Anxiety trait	
	*P* value	*R*	*P* value	*R*	*P* value	*R*	*P* value	*R*
Self-esteem		1.00	.56	–0.15	.04	–0.49	<.001	–0.72
Depressive characteristics				1	.21	0.31	.03	0.52
Anxiety characteristics						1.00	.02	0.55
Anxiety trait								1.00

**Table 5:** Correlation between preoperative psychological screening and postoperative pain.

Variables	Postoperative pain scores (inpatient)		Postoperative pain scores (after discharge)		Pain severity		Pain interference	
	*P* value	*R*	*P* value	*R*	*P* value	*R*	*P* value	*R*
Depressive characteristics	.96	0.01	.87	–0.44	.70	0.11	.09	0.46
Anxiety characteristics	.30	0.26	.22	–0.32	.47	–0.20	.09	0.46
Anxiety trait	.38	0.22	.64	–0.13	.78	–0.08	.02	0.58
Self-esteem	.34	–0.24	.51	0.18	.75	0.09	.01	–0.62

**Table 6:** Correlation between preoperative psychological screening and coping strategies.

Variables	Passive coping		Catastrophizing		Self-control	
	*P* value	*R*	*P* value	*R*	*P* value	*R*
Depressive characteristics	.12	0.41	.31	0.27	.49	–0.19
Anxiety characteristics	.03	0.55	.55	0.16	.99	–0.01
Anxiety trait	.89	0.04	.41	0.22	.32	–0.28
Self-esteem	.95	–0.02	.88	0.04	.21	0.34

## Discussion

### Principal Findings

The appearance of a chest wall deformity can decrease a patient’s psychological well-being such that self-perception is a major contributor to therapeutic decision making [5]. See comment in PubMed Commons below Furthermore, surgical care may cause severe stress or even psychological trauma [8]. Many investigators have shown that preoperative psychosocial factors such as anxiety increase postsurgical pain [2-4]. Moreover, patients undergoing thoracic surgery are prone to the development of chronic pain after surgery, which is often neuropathic and therefore more difficult to treat. Although psychological care is finally gaining attention and importance, many health care workers find it difficult to implement these challenging pain reduction strategies [26].

The primary aim was to introduce and evaluate the usefulness of eHealth technology for psychological screening purposes in an integrated surgical care model. Furthermore, 5 questionnaires were evaluated in assessing psychological variables (yellow flags such as depression, anxiety, and coping) involved in the transition from acute to persistent (subacute) pain in adolescent pectus patients. Finally, self-esteem was measured as an indirect parameter for pain persistence, as it is shown to be related with the incidence of yellow flags.

### eHealth Technology

eHealth is a relatively new practice in health care that includes electronic processes and communication. Although concerns are rising about user privacy and confidentiality, its importance is growing significantly [27,28]. We conducted this study to investigate its usefulness as part of a holistic surgical care process in adolescent pectus patients.

This study confirmed the easy accessibility of internet-based psychological screening questionnaires. Most patients quoted a low effort for questionnaire completion, reflecting patient compliance. Since we introduced an internet platform, patients can complete their tasks when and where they want, highlighting the importance of patient independency and responsibility.

In general, the implementation of Web-based questionnaires containing a preoperative psychological assessment can improve surgical outcomes for patients and their families if the optimal screening questionnaire depending on the surgical population is chosen.

### Psychological Variables and Type of Screening Questionnaire

It is well known that psychological characteristics play an important role in the development of persistent postsurgical pain; previous studies [29] have shown that trait anxiety increased pain after surgery [30-32]. In our data, preoperative depressive and anxiety states did not correlate with pain severity or pain intensity. This result is, however, somewhat inconsistent with the existing literature that shows that these states play a major role in chronification of pain [2-4,32]. One possible explanation is that psychological factors play a role in the development of chronic pain (defined as the persistence of pain for more than 3 months). The questionnaires used in this study protocol were, however, completed in the first week after discharge. Unfortunately, there was no long-term evaluation or a reassessment by retaking the applied screening battery. Consequently, further research is necessary to derive conclusions about chronic pain development and the extrapolation of the results to other patient populations.

Our results showed that anxious patients tended to engage more often in passive coping, which leads to maladaptive behaviors and cognitions about pain. This finding is in accordance with the literature on the chronification of pain. A study by Kaczynski et al [31] evaluated pain coping as a mediator of associations between anxiety and functional disability in adolescents with chronic pain. The authors indicated that relationships between anxiety systems and pain-related outcomes are complex. Their results showed that the association between anxiety and disability was mediated by passive coping [31].

There was no correlation between anxiety, depressive states, catastrophizing, and the experience of self-control. The overall mean catastrophizing score was low. This result is inconsistent with the literature on coping behaviors [33,34]. However, some authors have remarked on the concept of catastrophizing in children and adolescents [35-38]. One general remark should be made on the results of coping behavior and pain intensity and interference. The mean NRS score in the early postoperative phase was 1.89 (SD 0.82). The mean MPI pain severity score and interference after discharge were 1.88 (SD 0.78) and 3.20 (SD 0.69), respectively. These scores were low and could be attributable to the multidisciplinary follow-up before and after surgery. A pain sensation that is acceptable may indicate that the patient was able to cope with it. Conversely, because of the use of more adaptive coping styles, the pain was generally under control. Nevertheless, pain scores increased the first week after hospital discharge.

**Figure 5:**
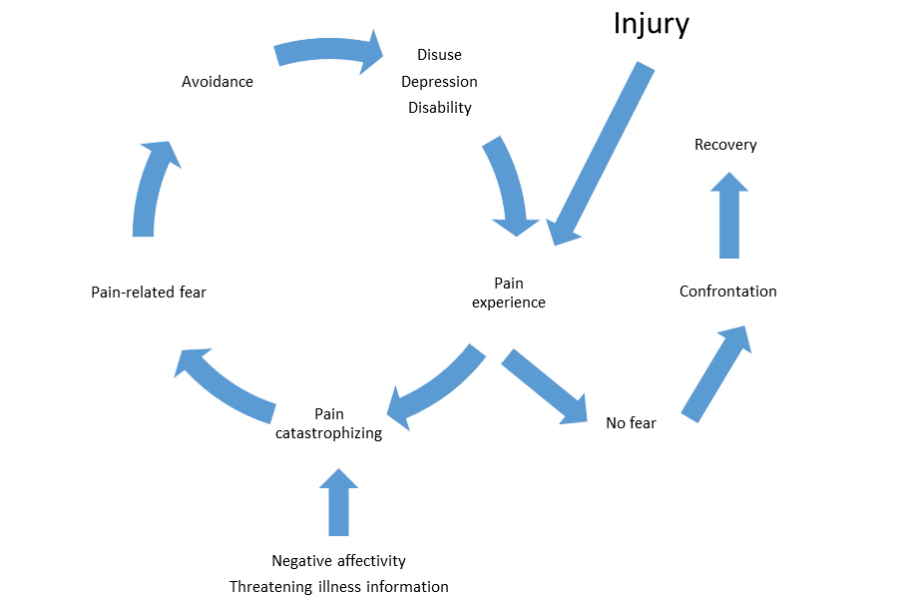
Fear avoidance model of pain by Vlaeyen and Linton [39].

We found several significant correlations: anxious predisposition and interference of pain, self-esteem and interference of pain, anxiety states and passive coping, self-esteem and anxiety measures, and depressive states and anxious predisposition. It is most likely that the relationships between anxiety, pain coping, and disability are bidirectional and contribute to a vicious circle of increasing pain-related disability as outlined in the fear avoidance model of pain by Vlaeyen and Linton [39] (Figure 5).

It is important to note that all study patients followed a specific postsurgical pathway that focused on pain (recovery). All patients had a preoperative consultation in the multidisciplinary pain center in which education about the eHealth system was provided. In addition to this practical information, the medical staff also provided information on acute, subacute, and chronic pain and self-management methods. During hospitalization, a multidisciplinary team of anesthesiologists, surgeons, physiotherapists, and nurses followed the postoperative rehabilitation protocol. Each provider could anticipate the concerns of the patients very quickly. This process of reassurance, encouraging questions, and cognitive reappraisal is important to reduce distress and anxiety, consistent with the findings of Sjoling et al [40]. This personal and specialized approach could be used therapeutically to address the experience of distress associated with hospitalization.

### Self-Esteem in Pectus Patients

Our results showed that preoperative anxiety is related to lower self-esteem, which is in accordance with the literature [22,41,42]. The mean self-esteem scores of this study sample were higher than the average Belgian levels of global self-esteem. This result is inconsistent with the expectation that pectus patients experience low self-esteem. Despite these findings, self-esteem played a role in the interference of postsurgical pain.

Self-esteem is an interesting measure in this population. There is a high comorbidity between depression and anxiety disorders. Low self-esteem is a transdiagnostic factor, for example, in both disorders. Improving self-esteem is an important treatment goal for therapy in depressive or anxious patients. Sowislo and Orth [41] evaluated the vulnerability and scar models of low self-esteem and depression, as well as low self-esteem and anxiety. The vulnerability model states that low self-esteem contributes to depression and anxiety, whereas the scar model states that low self-esteem is a consequence of depression and anxiety. The authors meta-analyzed the available longitudinal data. For depression, the findings supported the vulnerability model. For anxiety, the effects were relatively balanced; they found evidence for both theories. The authors speculated on why depression and anxiety were differentially linked to low self-esteem. They described, for example, that self-focused attention as a mediator is differentially related to depression and anxiety [41]. Additionally, many researchers further documented the concept of self-focus and suggested correlations between self-esteem and depressive and anxiety states [43-46].

We can question the use of the RSES in the measurement of self-esteem in children with pectus pathology. The RSES is a frequently used, short, and well-studied measure. In our study sample, all scores were relatively high. The study by Knudsen et al [47] reported the same ceiling effect in the use of the RSES as a measure of self-esteem. The purpose of their study was to assess the effects of surgical corrections of the pectus carinatum on health-related quality of life and self-esteem. Only one of 36 participants had low self-esteem (<15 points) according to the RSES before surgical correction, and self-esteem was within the normal range (>15 points) in all patients at the 6-month follow-up. This ceiling effect could be explained by the use of generic questions, resulting in high scores for self-esteem before surgery [47]. However, the RSES still remains a good measure of self-esteem [48], although some alternative multidimensional measures could be more sensitive.

Some authors [49,50] question the factor structure of the RSES. Current debate focuses on whether the RSES has a uni- or bidimensional structure (positive and negative self-esteem). Franck et al [17] evaluated the difference between the 1- and 2-factor models of the Dutch RSES questionnaire, and the questionnaire appears to represent a 1-dimensional construct of self-esteem, contaminated by the method effect primarily associated with the specific nature of the items. The predisposition to answer negatively worded items differently is associated with cognitive ability, age, cultural group membership, lower academic motivation, etc. The positive and reverse negative scores were 11.39 (SD 1.82) and 10.17 (SD 2.53), respectively (*P*=.06), indicating that patients answered consistently, independent of the positive or negative formulation of the items.

### Limitations

The limitations of our exploratory study need to be acknowledged. First, all questionnaires used were self-report instruments. Therefore, response bias may play a role, as results can vary due to small introspective abilities or socially desirable answering [51,52]. Second, we emphasize a potential time bias between hospital pain assessment and psychological evaluation via the MPI and CPQ. However, one may suggest an aberrant self-report from patients with a high postoperative pain score. A more precise evaluation of pain and coping technique could improve outcome variables. Furthermore, the reassessment of the preoperative questionnaires in the postoperative period could be of particular value. Nevertheless, minimal patient effort should be pursued. Third, the design of this proof-of-concept study may not use the questionnaire of choice in the assessment of self-esteem in adolescent pectus patients, as there was no significant difference in scores compared with those of healthy Belgians. To distinguish adolescent pectus patients with respect to self-esteem characteristics, a more sensitive and specific questionnaire is necessary.

### Conclusion

If caregivers involved in a surgical care process use innovative eHealth techniques as a simple, accessible psychological screening tool, along with adequate treatment if necessary, postoperative outcome parameters may further improve. As a fast, straightforward, and accessible instrument, an online platform can not only increase patient participation in rehabilitation but also alert the provider when yellow flags are present. To determine if this technique may be helpful in reducing postoperative pain, the length of hospital stay, and the development of chronic pain after surgery, more research is imperative.

## Abbreviations

APPI: Antwerp Personalized Pain Initiative
CPQ: Coping With Pain Questionnaire
CSQ: Coping Strategy Questionnaire
CT: computed tomography
HADS: Hospital Anxiety and Depression Scale
MIRP: minimally invasive repair of pectus
MPI: Multidisciplinary Pain Inventory
NRS: numeric rating scale
RSES: Rosenberg Self-Esteem Scale
STAI: State-Trait Anxiety Inventory
VAS: visual analog scale
