# Reference Correction: Preliminary Evaluation of a Web-Based Psychological Screening Tool in Adolescents Undergoing Minimally Invasive Pectus Surgery: Single-Center Observational Cohort Study

**DOI:** 10.2196/11608

**Published:** 2018-11-12

**Authors:** Davina Wildemeersch, Lisa Bernaerts, Michiel D’Hondt, Guy Hans

**Affiliations:** 1 Antwerp University Hospital Edegem Belgium

The authors of “Preliminary Evaluation of a Web-Based Psychological Screening Tool in Adolescents Undergoing Minimally Invasive Pectus Surgery: Single-Center Observational Cohort Study” (JMIR Ment Health 2018;5(2):e45) made two errors in the References section.

Rather than Rugo et al (2010), reference 6 should be as follows:

Brammer Jacobsen E, Thastum M, Jeppesen JH, Pilegaard HK. Health related quality of life in children and adolescents undergoing surgery for pectus excavatum. Eur J Pediatr Surg 2010;20:85-91.

Rather than Vigna et al (2006), reference 51 should be as follows:

Fan, X, Miller, BC, Park, K, Winward, BW, Christensen, M, Grotevant, HD, Tai, RH. An exploratory study about inaccuracy and invalidity in adolescent self-report surveys. Field Methods. 2006;18(3), 223-244.

The correction will appear in the online version of the paper on the JMIR website on November 12, 2018, together with the publication of this correction notice. Because this was made after submission to PubMed, PubMed Central, and other full-text repositories, the corrected article also has been resubmitted to those repositories.
